# The mediating role of self-efficacy in health literacy and chronic disease self-management in older individuals

**DOI:** 10.1186/s12877-026-07442-6

**Published:** 2026-04-23

**Authors:** Pınar Soylar, Besna Görnü

**Affiliations:** Health Sciences Faculty, Department of Nursing, Firat University, Elazig, 23119 Turkey

**Keywords:** Older adults, Chronic disease, Self-management, Health literacy, Self-efficacy, Mediation, Nursing

## Abstract

**Background:**

Chronic disease self-management (CDSM) is a vital component of healthy ageing, yet the interplay between its key determinants remains insufficiently understood. Health literacy and self-efficacy are widely recognized as important influences on older adults’ ability to manage chronic conditions; however, the pathways linking these factors have received limited attention. This study examined the mediating role of general self-efficacy in the relationship between health literacy and CDSM among older adults with chronic diseases in Türkiye.

**Methods:**

A cross-sectional survey was conducted with 183 individuals aged 65 years and older. Participants completed the Health Literacy Scale, the General Self-Efficacy Scale, and the Chronic Disease Self-Management Scale. Data were analysed using descriptive statistics, Pearson correlation, and mediation analysis via Hayes’ PROCESS macro with bootstrapping.

**Results:**

The mean age of participants was 71.6 years (SD = 6.9), and 51.4% were female. Health literacy, self-efficacy, and CDSM were all positively correlated (*p* < .001). Mediation analysis demonstrated that self-efficacy fully mediated the relationship between health literacy and CDSM, accounting for 64.7% of the total effect. These findings highlight the role of self-efficacy as a psychological mechanism through which health literacy influences self-management behaviours.

**Conclusions:**

Public health and health promotion initiatives targeting older adults should address both cognitive and psychosocial dimensions, aiming to strengthen self-efficacy alongside health literacy to support sustainable self-management and healthy ageing.

## Introduction

Population aging has become a significant global public health concern. According to the World Health Organization (WHO), the number of people aged 60 years and older is projected to reach 2.1 billion by 2050 [1]. Similarly, in Turkey, the proportion of older adults (aged 65 and above) increased from 8.2% in 2015 to 10.2% in 2022 and is expected to exceed 16% by 2040 [2]. As the global population continues to age, the prevalence of chronic non-communicable diseases (NCDs)—which represent the leading causes of morbidity and mortality among older adults—is also rising. This demographic and epidemiological shift highlights the urgent need for chronic disease self-management (CDSM) strategies that are both effective and specifically tailored to the unique needs of aging populations, who are disproportionately affected and require consistent, informed engagement over extended periods [3].

CDSM encompasses individuals’ ability to manage symptoms, adhere to treatment regimens, and implement lifestyle changes associated with chronic illnesses [4]. In this context, health literacy—defined as the capacity to obtain, process, and understand basic health information—plays a critical role [5, 6]. Although health literacy is often conceptualized as an individual skill, it is deeply influenced by social, cultural, and systemic factors, making disparities in health literacy a persistent barrier to health equity—particularly among older adults who frequently encounter cognitive, physical, and digital access limitations [7, 8]. While numerous studies have demonstrated that higher health literacy is linked to improved self-care behaviors, greater medication adherence, and lower healthcare utilization, the specific pathways through which health literacy facilitates better chronic disease self-management remain underexplored [5, 6]. In Türkiye, older adults often experience health system navigation challenges due to low education levels, insufficient primary care continuity, and lack of geriatric-specific programs, which can amplify the negative effects of low health literacy [9]. These structural disadvantages highlight the need for integrative approaches that address both cognitive and psychosocial dimensions of chronic disease care. One promising explanatory pathway identified in recent health behavior research is self-efficacy—a psychological construct referring to an individual’s belief in their ability to effectively manage health-related tasks and challenges.

Self-efficacy has emerged as a promising explanatory mechanism in recent health behavior research, defined as a psychological construct reflecting an individual’s belief in their capacity to effectively manage health-related tasks and challenges [10]. Self-efficacy, conceptualized by Bandura (1997) as an individual’s belief in their capability to execute specific actions, is a cornerstone of the Social Cognitive Theory [11]. It has been positively associated with health-promoting behaviors, including effective chronic disease management [10]. Among older adults, age-related physical, cognitive, and psychosocial changes may negatively affect both health literacy and self-efficacy, further complicating disease management. Therefore, it is essential to explore whether self-efficacy mediates the relationship between health literacy and CDSM in this vulnerable population.

Previous research suggests that self-efficacy does not merely function as an independent predictor of health behaviors, but also operates as an explanatory mechanism linking cognitive resources such as health literacy to behavioral outcomes. Several studies have demonstrated that self-efficacy mediates the relationship between health literacy and self-care behaviors, medication adherence, and chronic disease management, particularly among older adults and individuals with multimorbidity. In contrast, some studies have conceptualized self-efficacy as a moderating factor, indicating that the impact of health literacy on health behaviors may vary depending on individuals’ confidence in managing health-related challenges [12–15]. These mixed findings highlight the need for further empirical clarification of the functional role of self-efficacy, especially in aging populations where both health literacy and psychological resources may be compromised. However, in the present study, self-efficacy is conceptualized as a mediating mechanism rather than a moderating factor. This study aims to investigate the mediating role of self-efficacy in the association between health literacy and chronic disease self-management among older adults with chronic conditions in Türkiye. Understanding this relationship can inform the development of multi-level nursing interventions that are grounded in health promotion principles and aligned with global equity goals.

To address the aim of the study and better understand the interrelationships among health literacy, self-efficacy, and chronic disease self-management in older adults, the following research questions were formulated:

1. Is there a significant relationship between health literacy and chronic disease self-management in older adults?
2. Is there a significant relationship between self-efficacy and chronic disease self-management in older adults?
3. Is there a significant relationship between health literacy and self-efficacy in older adults?
4. Does self-efficacy mediate the relationship between health literacy and chronic disease self-management in older adults?

## Methods

### Study design and participants

This cross-sectional, correlational study was conducted to examine the mediating role of self-efficacy in the relationship between health literacy and chronic disease self-management among older adults. Based on a previous study conducted among individuals with chronic diseases, a power analysis was performed [12]. Taking into account a correlation coefficient of *r²* = 0.28 between health literacy and self-efficacy, the required sample size was calculated as 160, assuming a 95% confidence level (α = 0.05) and 80% power (1 − β = 0.80). To ensure robustness, an additional 23 participants were included beyond the calculated sample size, resulting in a final sample of 183 older adults. Given that the study was already powered at 80%, this increase further strengthened the reliability of the findings.

The study population consisted of individuals aged 65 years and older who were receiving care at hospital. Inclusion criteria were: being 65 years or older, having at least one diagnosed chronic disease, being able to communicate verbally, and voluntarily agreeing to participate. Individuals with severe cognitive impairment or diagnosed psychiatric disorders were excluded. A total of 183 participants were recruited using convenience sampling. Participants were informed about the aim, content, research procedures, and their right to withdraw from the study at any time. All eligible individuals provided written informed consent prior to completing the survey.

#### Ethics and declaration statements

The study was conducted according to the guidelines of the Declaration of Helsinki and approved by Fırat University Ethics Commission no. 2025/02–13. Human Ethics and Consent to Participate declarations: Written informed consent was obtained from all participants prior to completing the survey. Clinical trial number: not applicable.

### Data collection tools

#### General information form

This form included questions on age, gender, marital status, education, income level, perceived health status, and chronic disease diagnoses.

#### Health literacy scale for older adults

Health literacy was assessed using the Health Literacy Scale developed by Konopik et al. and adapted into Turkish by Genç et al. [16, 17]. This scale is specifically designed to measure health literacy in adult populations and consists of 18 items distributed across five subdimensions. Each item is rated on a 4-point Likert scale ranging from 1 (*strongly disagree*) to 4 (*strongly agree*), with higher scores indicating higher levels of health literacy. The total score ranges from 18 to 72. In the Turkish adaptation study, the scale demonstrated high internal consistency, with a Cronbach’s alpha coefficient of 0.92, indicating excellent reliability.

#### General self-efficacy scale

Self-efficacy was assessed using the General Self-Efficacy Scale developed by Jerusalem and Schwarzer to evaluate individuals’ belief in their ability to cope with challenges across various life domains. The scale consists of 10 items that measure confidence in overcoming difficulties, adhering to personal goals, and maintaining resilience, particularly in individuals with chronic diseases (CDs) [18]. Each item is rated on a 4-point Likert scale ranging from 1 (*not at all true*) to 4 (*exactly true*), yielding a total score between 10 and 40. Higher scores indicate greater perceived self-efficacy. The scale is unidimensional. The Turkish adaptation and validation were conducted by Yeşim Aypay, and the internal consistency reliability was reported as Cronbach’s alpha = 0.83, indicating good reliability for use in Turkish populations [19].

#### Chronic disease self-management scale

Chronic disease self-management was measured using the scale developed by Ngai et al. [20], which was adapted into Turkish by Öztürk et al. This instrument evaluates the self-management abilities of individuals with chronic illnesses across multiple psychosocial dimensions. Responses are rated on a 5-point Likert scale ranging from 1 (*Never*) to 5 (*Always*). The total score ranges from 23 to 115, with higher scores indicating better self-management skills. Items within the Treatment Adherence subscale are reverse scored. The scale is designed to capture both internalized perceptions and practical health-related behaviors, making it suitable for evaluating self-management in diverse chronic illness populations [21].

### Data analysis

Statistical analyses were performed using SPSS and Hayes’ PROCESS macro version 4.2 (Model 4). Descriptive statistics (means, standard deviations, frequencies, and percentages) were used to summarize sample characteristics and study variables. Pearson correlation analysis was conducted to examine relationships among health literacy, self-efficacy, and chronic disease self-management. Mediation analysis was performed to test whether self-efficacy mediated the relationship between health literacy and self-management. Indirect effects were evaluated using bootstrapping with 5,000 resamples and 95% bias-corrected confidence intervals. A p-value of less than 0.05 was considered statistically significant.

Self-efficacy was conceptualized as a mediating variable rather than a moderating variable based on both theoretical and empirical considerations. According to Social Cognitive Theory, self-efficacy represents a central cognitive mechanism through which knowledge and skills are translated into action. In this framework, health literacy provides individuals with the ability to access and understand health-related information, while self-efficacy determines the extent to which this knowledge is applied in daily self-management practices. Empirical studies in chronic disease populations have similarly modeled self-efficacy as a mediator linking health literacy to self-care behaviors, medication adherence, and disease management outcomes [11, 12, 14]. Therefore, the present study tested a mediation model to examine whether self-efficacy explains the pathway through which health literacy influences chronic disease self-management among older adults.

## Results

The study sample consisted of 183 older adults with chronic diseases (CDs). The mean age of participants was 71.63 years (SD = 6.98), and 51.4% were female. The majority had a primary school education (60.7%), while 25.7% had completed secondary school, and 13.6% had university-level education. Regarding income, 57.4% reported having a low income, 22.4% reported middle income, and 20.2% reported high income. In terms of self-rated health status, half of the participants (50.3%) reported poor health, while 27.9% rated their health as fair and 21.9% as good. The most frequently reported chronic conditions were hypertension (18.0%), Chronic Obstructive Pulmonary Disease (COPD) (14.2%), and diabetes (10.9%), and 50.8% of participants had two or more chronic diseases (Table 1).

**Table 1:** Demographic characteristics of older patients with chronic non-communicable diseases (*n* = 183)

Variable	*n*	%
Age (mean ± SD) 71.63 ± 6.98		
Gender		
Female	94	51.4
Male	89	48.6
Education Status		
Primary School	111	60.7
Secondary School	47	25.7
University	25	13.6
Incomel Level*		
High income	37	20.2
Middle income	41	22.4
Lower income	105	57.4
Health Status*		
Good	40	21.9
Fair	51	27.9
Poor	92	50.3
Chronic Diseases		
Diabetes	20	10.9
Hypertension	33	18.0
COPD	26	14.2
Circulatory system disease	11	6.0
Number of CDs: 2 or more	93	50.8

*Self reported; *CDs* Chronic Diseases, *COPD* Chronic Obstructive Pulmonary Disease

Table 2 presents the means, standard deviations, and pearson correlation coefficients among the main study variables. The mean scores were 45.89 (SD = 11.15) for health literacy, 26.59 (SD = 6.33) for self-efficacy, and 64.49 (SD = 7.77) for chronic disease self-management. Bivariate correlation analysis showed that chronic disease self-management was significantly and positively correlated with both health literacy (*r* = .347, *p* < .001) and self-efficacy (*r* = .380, *p* < .001). Moreover, a strong positive correlation was found between health literacy and self-efficacy (*r* = .781, *p* < .001), indicating that all key variables were significantly associated at the 0.01 level.

**Table 2:** Mean scores and correlation matrix of main study variables (*n* = 183)

Variable	Mean (SD)	Health Literacy	Self-efficacy	CDSM
Health Literacy	45.89 (11.15)	1	0.781**	0.347**
Self-efficacy	26.59 (6.33)	0.781**	1	0.380**
CDSM	64.49 (7.77)	0.347**	0.380**	1

*SD* Standard deviation; **p < .01; *CDSM* Chronic disease self-management

The mediation model examined whether general self-efficacy mediated the relationship between health literacy and chronic disease self-management. The mediation analysis was conducted without adjusting for demographic covariates. The model focused on testing the theoretical relationships among health literacy, self-efficacy, and chronic disease self-management. The total effect of health literacy on chronic disease self-management was significant (*β* = 0.225, 95% CI [0.1209, 0.3294]). The indirect effect, mediated by self-efficacy, was *β* = 0.145 (95% CI [0.0202, 0.2822]), accounting for approximately 64.7% of the total effect. The direct effect of health literacy on chronic disease self-management became nonsignificant when self-efficacy was included in the model (*β* = 0.080, 95% CI [–0.0721, 0.2314]), suggesting a full mediation effect (Table 3). These findings indicate that general self-efficacy plays a key mediating role in the pathway from health literacy to chronic disease self-management among older adults.

**Table 3:** Direct, indirect, and total effects of predictor variables in the model

	Pathway	β	SE	95% CI	Proportion
Direct effect	(HL → CDSM)	0.080	0.077	[–0.0721, 0.2314]	35.33%
Mediated effect	(HL → SE → CDSM)	0.145	0.066	[0.0202, 0.2822]	64.67%
Total effect	(HL → CDSM)	0.225	0.053	[0.1209, 0.3294]	100%

*HL* Health Literacy, *SE* Self Efficacy, *CDSM* Chronic disease self-management, *CI* Confidence interval, Bootstrap sample size = 5,000, *CI* Confidence Interval, *SE* Standard Error. The indirect effect is significant since the 95% CI does not include zero. *p* < .05

**Fig. 1:**
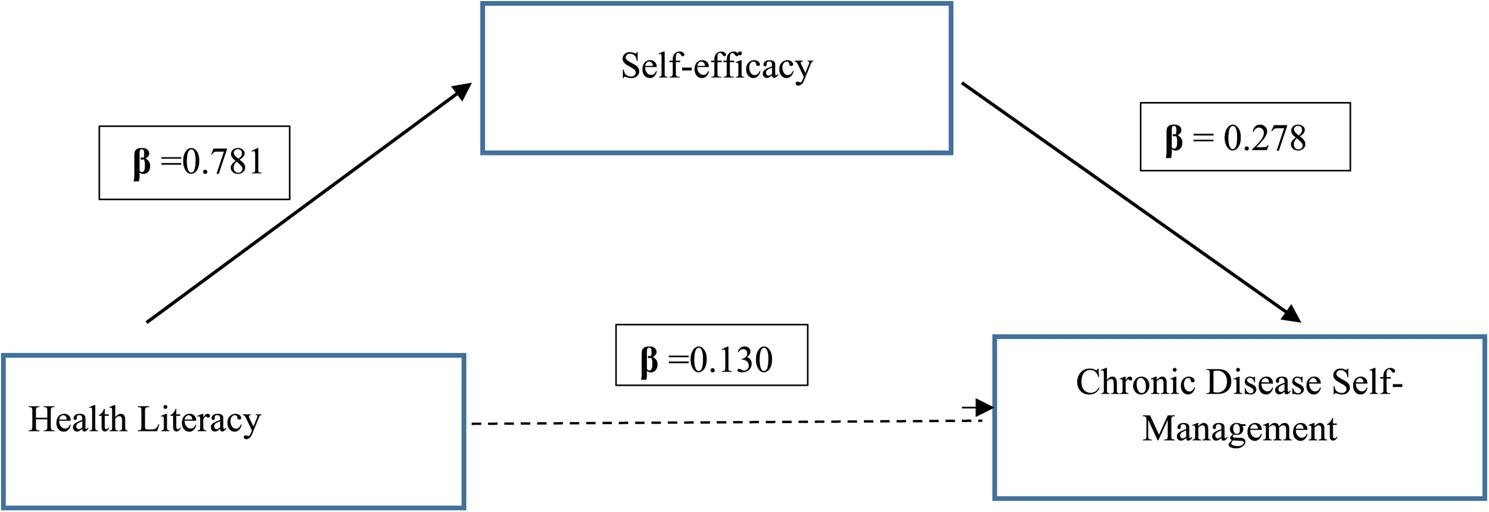
Mediation model: the role of self-efficacy. Path coefficients (β) are based on linear regression models

Figure 1 presents the mediation model examining the effect of health literacy on chronic disease self-management (CDSM) through self-efficacy. The standardized regression coefficient from health literacy to self-efficacy was significant (β = 0.781, *p* < .001), indicating a strong positive association. In the second regression model, self-efficacy significantly predicted CDSM (β = 0.278, *p* = .012), while the direct effect of health literacy on CDSM became nonsignificant (β = 0.130, *p* = .238) when self-efficacy was included in the model.

## Discussion

The present study contributes to the growing literature on chronic disease self-management in older adults by emphasizing the combined influence of health literacy and psychological resources. Chronic disease self-management is widely recognized as a complex behavioral process that requires not only access to health-related information but also the capacity to apply this information consistently in daily life [4, 20]. In older populations, this process may be further complicated by age-related physical limitations, cognitive changes, and increasing disease burden [3, 8].

Health literacy has been consistently identified as a key determinant of effective self-management, enabling individuals to understand treatment instructions, navigate healthcare systems, and engage in preventive behaviors [5, 6]. Previous studies conducted among older adults have shown that limited health literacy is associated with poorer disease control, lower medication adherence, and reduced engagement in health-promoting behaviors [7, 8, 21]. Similarly, researches from Türkiye indicates that older individuals often experience difficulties in accessing and using health information due to educational, socioeconomic, and system-level barriers [9, 22]. Evidence from studies conducted in Türkiye further suggests that the relationship between health literacy and self-care behaviors is often indirect, operating through empowerment-related constructs such as perceived control and confidence in managing illness [23]. These findings support the notion that health literacy constitutes an essential, yet insufficient, foundation for chronic disease self-management.

Beyond cognitive capacity, self-efficacy has been identified as a central psychological factor influencing individuals’ ability to manage chronic conditions [10, 24]. Self-efficacy reflects individuals’ beliefs in their capacity to perform behaviors necessary to achieve desired health outcomes and has been shown to predict adherence, persistence, and coping in chronic illness contexts [11, 25]. Among older adults, declining physical strength, multimorbidity, and dependence on others may negatively affect self-efficacy, thereby limiting active engagement in self-management behaviors even when health-related knowledge is present [26, 27].

One of the key contributions of this study is the demonstration of self-efficacy as a mediating mechanism between health literacy and chronic disease self-management. This finding suggests that health literacy may influence self-management indirectly by strengthening individuals’ confidence in their ability to manage health-related tasks. Similar mediation effects have been reported in previous studies, where self-efficacy explained the relationship between health literacy and self-care behaviors, medication adherence, and disease management outcomes [12–15]. These findings indicate that knowledge alone does not automatically translate into action unless individuals believe they are capable of applying that knowledge effectively.

This mediating role can be interpreted within the framework of Social Cognitive Theory, which posits that behavior change is driven by reciprocal interactions among cognitive resources, personal beliefs, and environmental factors, with self-efficacy serving as a core mechanism linking knowledge to action [11]. In line with this theory, studies have shown that interventions enhancing both health literacy and self-efficacy lead to more sustainable improvements in chronic disease outcomes compared to information-based approaches alone [26, 28]. The full mediation observed in this study further reinforces the idea that self-efficacy plays a decisive role in enabling older adults to transform health information into consistent self-management practices.

The findings are also consistent with research demonstrating that self-efficacy interacts with broader psychosocial factors, such as social support and illness perception, in shaping chronic disease management behaviors [12, 29]. Older adults who lack confidence in their ability to manage symptoms or adhere to treatment regimens may experience fear of mismanagement or loss of autonomy, which can hinder the practical use of health information [14]. In this context, self-efficacy functions as a psychological bridge between knowing and doing, particularly in aging populations where vulnerability and dependency are more pronounced.

From a nursing and public health perspective, these findings highlight the importance of adopting comprehensive, empowerment-oriented approaches to chronic disease management. While improving health literacy remains a critical goal, interventions that fail to address psychological readiness and confidence may yield limited behavioral change [6, 10]. Nurse-led self-management programs, motivational support, and skills-based interventions that explicitly target self-efficacy have been shown to improve adherence, quality of life, and disease control among individuals with chronic conditions [20, 24, 25]. In Türkiye, nurse-led self-management interventions have demonstrated positive effects on self-care behaviors and disease control, particularly when structured models and tele-nursing approaches are used, underscoring the practical relevance of empowerment-based strategies in chronic disease management [30]. Integrating such approaches into routine care for older adults may therefore enhance the effectiveness and sustainability of self-management interventions.

## Conclusion

This study highlights the critical role of self-efficacy as a psychological mechanism through which health literacy influences chronic disease self-management among older adults. The findings indicate that improving health literacy alone may not be sufficient to promote sustained self-management behaviors unless older individuals also feel confident in their ability to apply health-related knowledge in daily life. From an academic perspective, this study contributes to the literature by clarifying the functional role of self-efficacy as a mediating factor in the relationship between health literacy and self-management, thereby strengthening theoretical models grounded in Social Cognitive Theory. By focusing on older adults, the findings also address an important gap in aging and chronic disease research, where cognitive and psychosocial determinants are often examined in isolation.

In terms of health policy, the results underscore the need for integrated health promotion strategies that go beyond information dissemination. Policies aimed at improving outcomes in aging populations should incorporate empowerment-based components that enhance older adults’ confidence, autonomy, and perceived control over chronic disease management. Embedding self-efficacy–oriented approaches into national aging and chronic care frameworks may contribute to more sustainable and equitable health outcomes. For healthcare professionals, particularly nurses working with older adults, the findings emphasize the importance of adopting holistic care approaches that address both cognitive and psychosocial dimensions of self-management. Nurse-led interventions that combine health education with motivational support, skills training, and individualized goal-setting may be particularly effective in fostering long-term engagement in chronic disease self-management among older adults. Overall, this study suggests that strengthening self-efficacy alongside health literacy may be a key strategy for improving chronic disease self-management and supporting healthy aging in older populations.

### Limitations

Several limitations should be considered when interpreting the findings. First, demographic and clinical variables such as age, gender, education level, and number of chronic conditions were not included as covariates in the mediation model. Therefore, potential confounding effects cannot be fully ruled out. Future studies using larger samples should incorporate these variables to test the robustness of the mediation model.

## Data Availability

The data that support the findings of this study are available from the corresponding author upon reasonable request.
